# PIK3R3 inhibits cell senescence through p53/p21 signaling

**DOI:** 10.1038/s41419-020-02921-z

**Published:** 2020-09-24

**Authors:** Qianzhi Chen, Xuling Sun, Xuelai Luo, Jing Wang, Junbo Hu, Yongdong Feng

**Affiliations:** 1 Department of GI Cancer Research Institute, Tongji Hospital, Tongji Medical College, Huazhong University of Science and Technology, Wuhan, Hubei China; 2 Department of Breast and Thyroid Surgery, Union Hospital, Tongji Medical College, Huazhong University of Science and Technology, Wuhan, Hubei China; 3 Department of Immunology, Basic of Medicine, Tongji Medical College, Huazhong University of Science and Technology, Wuhan, Hubei China

**Keywords:** Cancer, Cell biology

## Abstract

Cellular senescence is a stress response of human cells that removes potentially harmful cells by initiating cell cycle arrest. Inducing senescence of tumor cells may be an effective tumor-inhibiting strategy. In this study we found that PIK3R3 could inhibit the cell senescence of colorectal cancer cells and promote cell proliferation through the p53/p21 signal pathway. PIK3R3 could bind to p53 and inhibit the binding of p53 to the p21 gene promoter region, and thus affecting the transcriptional activity of p21 gene. Our study has provided new evidence of the role of PIK3R3 in p53 regulation and inhibition of PIK3R3 may be one of the potential targets of tumor therapy.

## Introduction

Colorectal cancer (CRC) is a common malignancy of the gastrointestinal tract, with its mortality rate ranking the third of all types of cancer around the world^1^. CRC greatly reduces life quality and causes heavy economic and societal burden. With the development of human societies and the changes in dietary habit, both the incidence and the mortality rate of CRC are increasing year by year^2^. Though in recent years the operative procedures for CRC treatment are being continuously improved and new drugs being developed, the prognosis of CRC patients with CRC is still far from optimal^3^. Discovering the unknown molecular mechanisms of CRC for the development of new potential treatment modalities of CRC is therefore the focus of our current research.

PIK3R3 is one of the regulatory subunits of phosphoinositide 3-kinase, which notably affects cell genesis, proliferation, differentiation, apoptosis, and metabolism^4–9^. Xia et al. found that PIK3R3 can directly bind to retinoblastoma (Rb) protein through its N-terminal 24 highly fidelity amino acids (N24), thereby regulating cell cycle. Our previous study found that PIK3R3 can bind to proliferating cell nuclear antigen (PCNA) to promote cell proliferation^10^, indicating that PIK3R3 can bind to Rb and PCNA to synergistically regulate the cell cycle.

p53 known as Gene Guard is an important tumor suppressor gene in the human body. Wild-type p53 maintains normal cell growth and inhibits tumor proliferation. p21, first discovered and named by Harper et al., can inhibit the activity of cyclin-dependent kinase (CDK), also known as CDKN1A (cyclin-dependent kinase inhibitor 1)^11^. El-Deiry et al.^12^ found that p21 is a downstream gene of p53, and its expression level is regulated by p53 (ref. ^12^). There are binding sites for interaction with other proteins on two conserved domains of p21 protein, including N-terminal and C-terminal cyclin-binding sites, C-terminal PCNA-binding sites, etc.^13^. p21 protein binds to the CDK/cyclin complex and inhibits its activity, thereby inhibiting Rb phosphorylation and then arresting the cell cycle^11,14^.

Our previous study found that p53 can promote the expression of miR-148b by binding to its promoter, while miR-148b can act on the 3′-UTR region of PIK3R3 to inhibit its expression, thereby inhibiting cell proliferation, tumor formation and progression^10^. In cell lines experiments, we found that PIK3R3 protein could bind to p53 protein and the intervention of PIK3R3 could significantly affect cell senescence. We know that the p53/p21 signaling axis is an important pathway for cell senescence regulation^15–18^. Based on the above clues, this study will further clarify how PIK3R3 affects cell senescence through the p53/p21 pathway.

## Results

### PIK3R3 is overexpressed and p21 is underexpressed in CRC

**Fig. 1:**
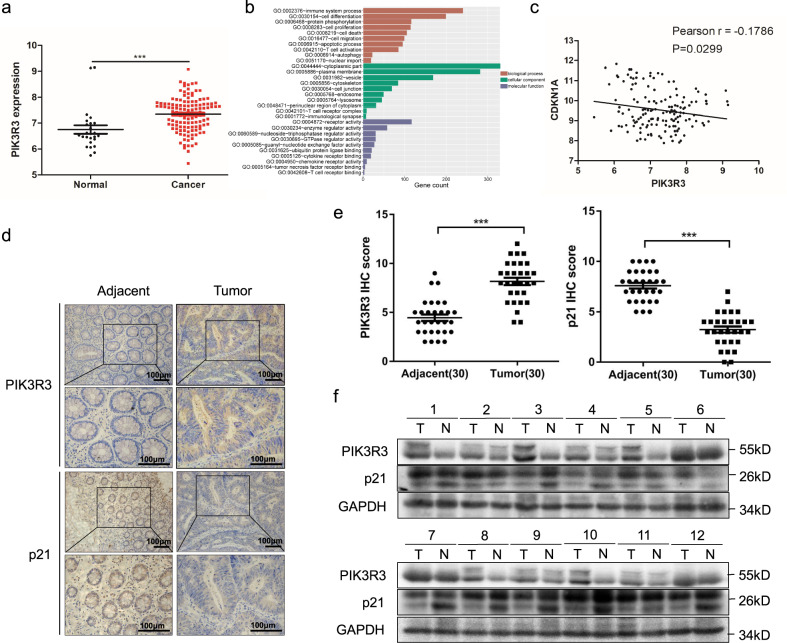
PIK3R3 is overexpressed and p21 is underexpressed in colorectal cancer. **a** Expression analysis of PIK3R3 in CRC tissues and normal intestinal tissues from a dataset (GSE21510) of GEO database. **b** Gene ontology (GO) analysis of PIK3R3 co-expression genes**. c** Correlation analysis of PIK3R3 and p21 in CRC tissues from the dataset (GSE21510). **d** IHC staining of typical samples from CRC and adjacent tissues. **e** Quantification of PIK3R3 and p21 expression in tumor groups and normal epithelial groups. **f** The protein levels of PIK3R3 and p21 in CRC and adjacent normal tissues. These numeral results were displayed as mean ± SD, *p < 0.05, **p < 0.01, ***p < 0.001, *t*-test.

We first retrieved the Gene Expression Omnibus (GEO) databases and analyzed CRC datasets. Then we found that the expression of PIK3R3 in human normal intestinal tissues is significantly lower than that in CRC tissues (Fig. 1a). Gene Ontology (GO) analysis of co-expression genes was performed, and the results showed that PIK3R3 expression is involved in several important biological processes, such as proliferation, cell death, migration, immune system process, and protein phosphorylation. Cellular component data of PIK3R3-related genes were related to cytoplasmic part, plasma membrane, endosome, T cell receptor complex, and immunological synapse. Molecular function data showed that PIK3R3 expression was associated with receptor activity, enzyme regulator activity, ubiquitin protein ligase binding, and cytokine receptor binding (Fig. 1b). GO analysis showed PIK3R3 mainly functions in cytoplasm and takes part in cell proliferation regulation. Therefore, we used bioinformatics analysis to explore the possible relationship between PIK3R3 and proliferation signal pathway genes. Correlation analysis showed PIK3R3 expression was negatively correlated with p21 expression in CRC dataset (Fig. 1c).

From immunohistochemical staining data we found that PIK3R3 expression level was increased and p21 was decreased in tumor samples in contrast to adjacent tissues (Fig. 1d). Matching analysis of immunohistochemistry (IHC) scores showed that the PIK3R3 protein level was higher and the p21 protein level was lower in tumor tissues (*p* < 0.001, Fig. 1e). To further validate these results, we measured PIK3R3 and p21 protein expression in 12 pairs of CRC specimens and adjacent epithelial tissues via western blot. PIK3R3 expression was significantly higher in tumor samples than in normal epithelial tissues, while p21 expression was correspondingly lower in tumor samples than in normal epithelial tissues in 10 out of 12 pairs (83.3%, Fig. 1f). These data indicated that PIK3R3 is highly expressed in CRC tissues and negatively correlated with the p21 expression level.

### PIK3R3 regulates p21 expression in a p53-dependent manner

**Fig. 2:**
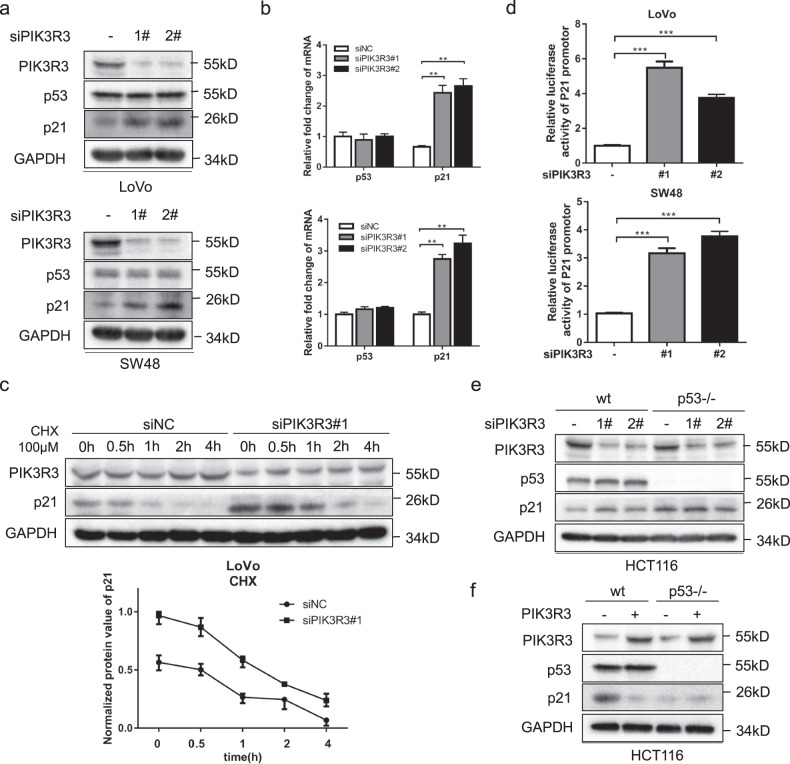
PIK3R3 regulates p21 expression in the p53-dependent way. **a** Western blot (WB) of PIK3R3, p53, and p21 both in LoVo and SW48 cells transfected with siNC, siPIK3R3(#1,#2). **b** qPCR assays of PIK3R3, p53, and p21 mRNA in LoVo (up panel) and SW48 (down panel) cells transfected with siNC, siPIK3R3 (#1,#2). These data were normalized to corresponding GAPDH. **c** LoVo cells transfected with siNC or siPIK3R3#1 were treated with CHX(100 μM) in different time lengths (0, 0.5, 1, 2, and 4 h). WB showed the change trend of p21 protein over time. **d** Luciferase activities of p21 promotor in LoVo (left panel) and SW48 (right panel) cells transfected with p21-luc and siNC, siPIK3R3(#1,#2). **e**, **f** WB of PIK3R3, p53, and p21 in HCT116 p53−/− cells transfected with siNC, siPIK3R3 (#1, #2), or with PIK3R3-expressing plasmid. Every assay repeated three times, and these numeral results were displayed as mean ± SD, *p < 0.05, **p < 0.01, ***p < 0.001, *t*-test.

As we know, p53 is known as an important transcription factor and can regulate the promotor of p21. We measured the p53 and p21 expression levels after knockdown of PIK3R3 in LoVo and SW48 cells. We found that p21 expression increased both on protein and mRNA levels while there was no significant change in p53 expression (Fig. 2a, b). Furthermore, we confirmed that PIK3R3 knockdown did not affect the protein degradation process of p21 (Fig. 2c). The luciferase assays showed that p21 promoter luciferase activity increased by silencing PIK3R3 (Fig. 2d). These data indicated PIK3R3 might regulate p21 by affecting the transcription activity of p21. To verify whether the promoter activation of p21 is dependent on p53, we selected HCT116 p53−/− cells, i.e. p53-null cells originating from HCT116 wild-type cells. Neither knocking down or overexpressing PIK3R3 induced significant change of p21 protein expression in HCT116 p53−/− cells compared with HCT116 wt cells (Fig. 2e, f), supporting the regulation of p21 transcription by PIK3R3 in a p53-dependent manner.

### Knockdown of PIK3R3 promotes cell senescence and inhibits cell proliferation

**Fig. 3:**
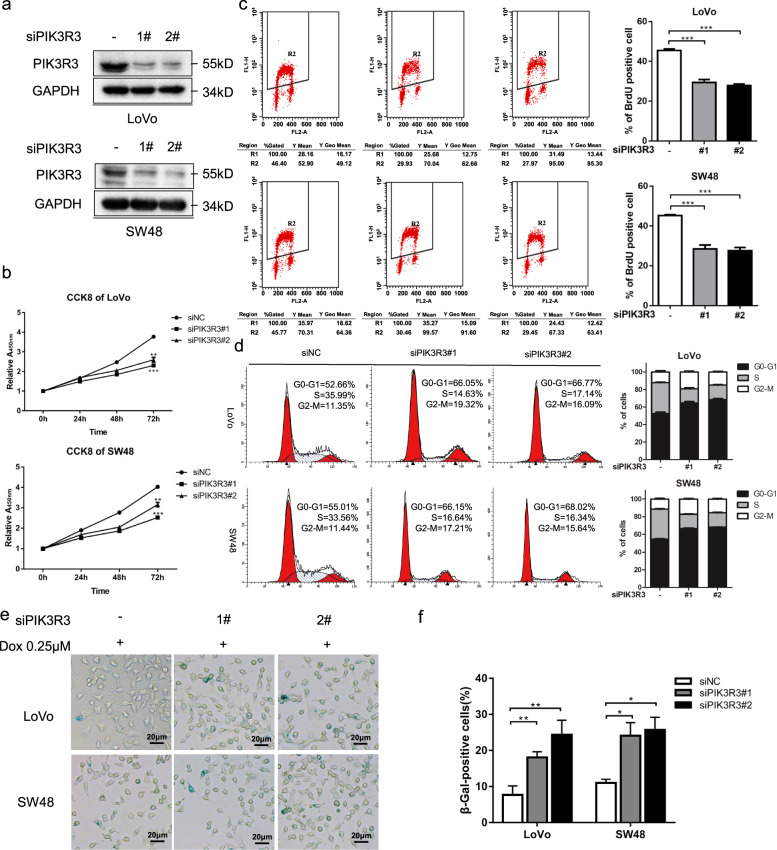
Knockdown of PIK3R3 promotes cell senescence and inhibits cell proliferation. **a** WB of PIK3R3 and p21 both in LoVo and SW48 cells transfected with siNC, siPIK3R3#1, and siPIK3R3#2. **b** CCK8 assays of LoVo and SW48 cells transfected with PIK3R3-specific siRNA. **c** BrdU-positive cells percent of PIK3R3-knockdown LoVo and SW48 cells. **d** Cell cycle distribution of LoVo and SW48 cells transfected with siNC, siPIK3R3#1, and siPIK3R3#2 was measured by flow cytometry. **e**, **f** SA-β-gal staining of PIK3R3-knockdown LoVo or SW48 cells after treatment of Dox (0.25 μM, 48 h). Every assay repeated three times, and these numeral results were displayed as mean ± SD, *p < 0.05, **p < 0.01, ***p < 0.001, *t*-test.

We continued to perform the p21 downstream phenotype experiments to determine the extent of PIK3R3’s influence on cell senescence and proliferation. We used PIK3R3 knockdown model produced by transfecting LoVo and sw48 cell lines with two siRNA (Fig. 3a). CCK8 assay to detect cell proliferation showed that knocking down PIK3R3 decreased the cell growth rate in LoVo and SW48 cells (Fig. 3b). The BrdU-positive cells ratio in PIK3R3 knockdown cells was also less than that in negative control cells (Fig. 3c). We applied flow cytometry to observe cell cycle distribution, and the results showed PIK3R3 knockdown decreased the percentage of cells in the G0/G1 phase but increased those in the S phase (Fig. 3d).

We observed that the PIK3R3 knockdown cells showed enlarged and flattened shape that was typical senescent morphology (data not shown). It is well known that cell senescence is a special cell state and is characterized by slow proliferation and cell cycle arrest. SA-β-gal (one senescence biomarker) staining assay was used to detect cell aging, and the results showed that the proportion of the SA-β-gal staining-positive cells in PIK3R3 knockdown cells were increased in contrast to that in negative control cells (Fig. 3e, f). Senescent cells could acquire the senescence-associated secretory phenotype (SASP) that consists of a highly complex mixture of secreted cytokines, chemokines, growth factors, and proteases^19,20^. We used qPCR to test SASP, and the data showed that the mRNA expression of CCL20, IL1β, IL6, and IL8 increased in PIK3R3 knockdown cells compared with negative control cells in LoVo and SW48 cell lines (Supplementary Fig. S1a, b). Our data suggest that PIK3R3 plays an important role in the regulation of cell senescence and proliferation in CRC cells.

### PIK3R3 combines with p53 and enhances its transcriptional activity

**Fig. 4:**
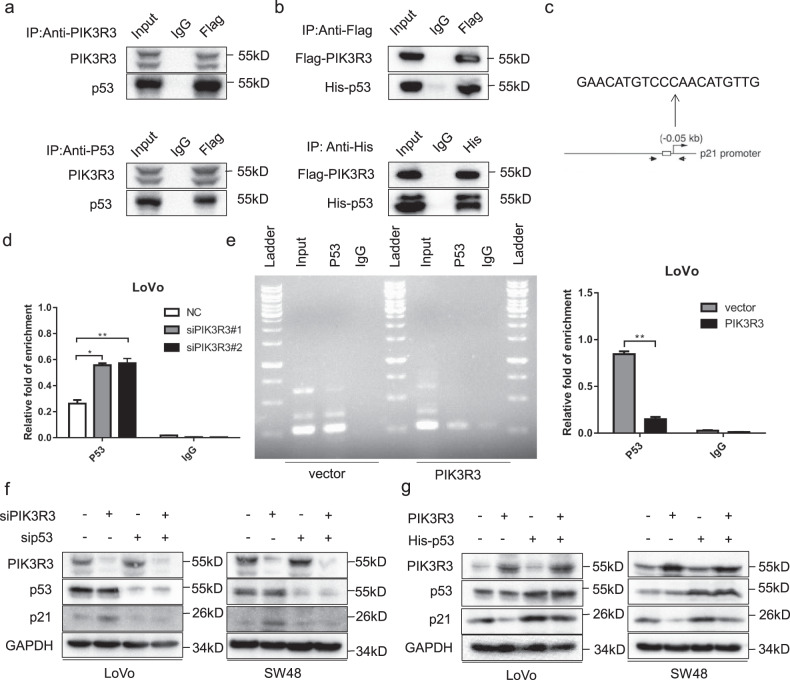
PIK3R3 combines p53 and enhances its transcriptional activity. **a** Immunoprecipitation assays were performed for LoVo cells with anti-PIK3R3 or anti-p53 antibodies. **b** Immunoprecipitation assays with antibodies against Flag or His in LoVo tranfected with Flag-PIK3R3 or His-p53. **c**, **d** ChIP assays of p53 binding to the CDKN1A promoter region in LoVo transfected with siPIK3R3#1. **e** ChIP assays of p53 binding to the CDKN1A promoter region in LoVo transfected with PIK3R3-expressing plasmid. **f** WB of PIK3R3, p53, and p21 in LoVo and SW48 cells transfected with siPIK3R3#1 or/and sip53. **g** WB of PIK3R3, p53, and p21 in LoVo and SW48 cells transfected with PIK3R3 plasmid or/and His-p53 plamid. Every assay was repeated three times, and these numeral results were displayed as mean ± SD, *p < 0.05, **p < 0.01, ***p < 0.001, *t*-test.

Although PIK3R3 did not affect the protein expression level of p53, we found that p21 was regulated via p53-dependent way. Therefore, we used co-immunoprecipitation (Co-IP) experiments in LoVo cells to verify the PIK3R3–p53 interaction, and found that PIK3R3 and p53 could precipitate with each other (Fig. 4a). In LoVo cells co-transfected with Flag-PIK3R3 and His-p53 we observed the same results (Fig. 4b). We used a confocal microscope to show the co-localization of PIK3R3 and p53, and found that PIK3R3 and p53 were mainly co-localized in the cytoplasm in 293t cells transferred with His-P53 and Flag-PIK3R3 plasmids (Fig. S2a). In LoVo cells we knocked down PIK3R3, then the distribution of p53 increased in the nucleus and decreased in the cytoplasm, while with a high expression of PIK3R3 the distribution of p53 decreased in the nucleus and increased in the cytoplasm (Supplementary Fig. S2b, c). p53 is a major transcription factor of p21, and p53 plays an important role in p21 transcriptional activation (Fig. 4c). To further investigate how PIK3R3 affected p21 transcriptional activity, chromatin immunoprecipitation (ChIP) experiments were performed to detect the binding of p53 on the p21 promoter region in PIK3R3 knockdown or overexpression LoVo cells (Fig. 4d, e). The results indicated that the binding of p53 to p21 promoter increased in PIK3R3 knockdown cells, and decreased in PIK3R3-overexpressing cells.

In order to further demonstrate the pivotal role of p53 in PIK3R3 overexpression-induced p21 suppression, we knocked down PIK3R3 in LoVo and sw48 cells with or without p53 knockdown, and then tested changes of p21 levels. Down-expression of PIK3R3 notably increased the protein expression of p21, which could however be reversed by knocking down p53 (Fig. 4f). Correspondingly, overexpressing p53 reversed the low expression of p21 induced by high expression of PIK3R3 (Fig. 4g).

Due to extensive chromatin remodeling, senescent cells mostly tend to form senescence-associated heterochromatin foci (SAHF), and HP1α accumulation is one of the typical signs of SASF^21,22^. To further solidify the PIK3R3/p53/p21 axis in the regulation of cell senescence, we knocked down PIK3R3 in LoVo and sw48 cells with or without p21 knockdown, then tested changes of HP1α levels. Down-expression of PIK3R3 notably increased the protein expression of HP1α, which could however be reversed by knocking down p21 (Supplementary Fig. S3a). Correspondingly, overexpressing p21 reversed the low expression of HP1α induced by high expression of PIK3R3 (Supplementary Fig. S3b).

Taken together, PIK3R3 binds to p53 and inhibits p53 into the nucleus, thereby inhibits p53-dependent transcriptional activation of p21, which induces cell senescence suppression (Supplementary Fig. S4).

### PIK3R3 knockdown suppresses cell proliferation and promotes cellular senescence in vivo

**Fig. 5:**
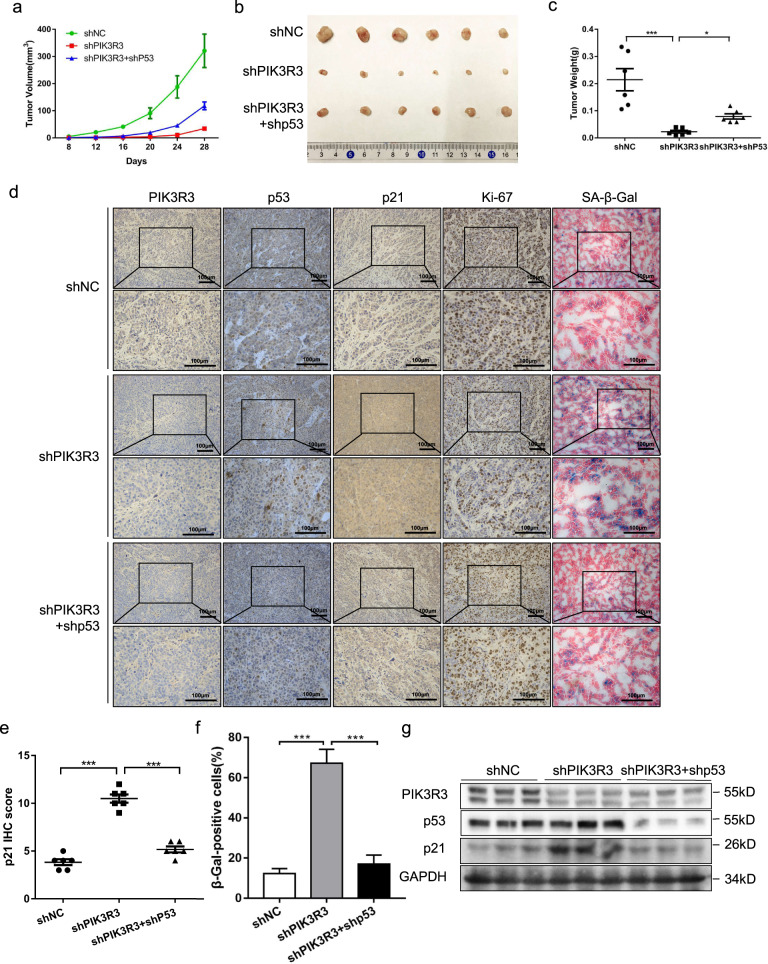
PIK3R3 knockdown suppresses cell proliferation and promotes cell senescence in vivo. **a** Tumor volume of three groups were measured over time. **b** Tumor gross observation of three groups (shNC, shPIK3R3, and shPIK3R3 + shp53). **c** Tumor weight of three groups (vector, PIK3R3, and PIK3R3 + p53). **d** IHC assays of PIK3R3, p53, p21, Ki-67, and β-Gal from three groups. **e** Quantification of p21 expression from three groups. **f** Quantification of the proportion of β-Gal-positive cells from three groups. **g** WB of PIK3R3, p53 and p21 in three different groups. These numeral results were displayed as mean ± SD, *p < 0.05, **p < 0.01, ***p < 0.001, *t*-test.

To confirm the role of PIK3R3 in cell proliferation and cellular senescence of CRC cells in vivo, the nude mice xenograft tumor model was constructed. First we injected three stable expressed cell lines (LoVo cells transfected with shNC, shPIK3R3, and shPIK3R3 + shp53, respectively) into the subcutaneous tissues of nude mice (each group 6 mice, 1 × 10^6^ cells every mouse). The size of the subcutaneous xenografts was measured after 1 week, and then we measured the volume of the tumor every 4 days. The data were further made into a tumor volume growth graph, which showed that the tumors of the shPIK3R3 group grew more slowly than the shNC group, but the shPIK3R3 + shp53 group grew faster than the shPIK3R3 group (Fig. 5a). After 1 month of tumor growth we sacrificed all nude mice and harvested the tumor tissues. Tumor weight was measured in order to create a chart, and the results provided an expected conclusion (Fig. 5b, c). The results of immunohistochemistry revealed that the proportion of Ki-67 staining cells in the shPIK3R3 group was lower than that in the shNC group, and the proportion of Ki-67 staining cells in the shPIK3R3 + shp53 group was higher than that in the shPIK3R3 group, while the percentage of β-Gal-positive cells was exactly the opposite to Ki-67 staining cells (Fig. 5d, f). The expression level of p21 increased in the shPIK3R3 group compared with the shNC group, and decreased in the shPIK3R3 + shp53 group compared with the shPIK3R3 group (Fig. 5d, e). Mitochondrial dysfunction mediated elevated levels of reactive oxygen species in senescent cells can cause accumulation of lipofuscin, which can be demonstrated by Sudan Black B (SBB) in immunohistochemical staining assays^23,24^. Another hallmark of cellular senescence is the formation of SAHFs, which are riched in heterochromatin protein 1 (HP1)^21,22^. So we used these two additional markers to show cellular senescence in subcutaneous xenograft tumor cells. The staining level of HP1α and Lipofuscin (SBB staining) increased in the shPIK3R3 group compared with the shNC group, and decreased in the shPIK3R3 + shp53 group compared with the shPIK3R3 group (Supplementary Fig. S5a). The protein levels of PIK3R3, p53, and p21 from three different groups were measured by western blotting, and we obtained the results similar to the data in vitro (Fig. 5g). The above data showed that silencing PIK3R3 suppressed cell proliferation and promoted cell senescence of subcutaneous xenograft tumor tissues in the mice model, which could be reversed by p53 depletion.

### Overexpressed PIK3R3 promotes cell proliferation and inhibits cellular senescence in vivo

**Fig. 6:**
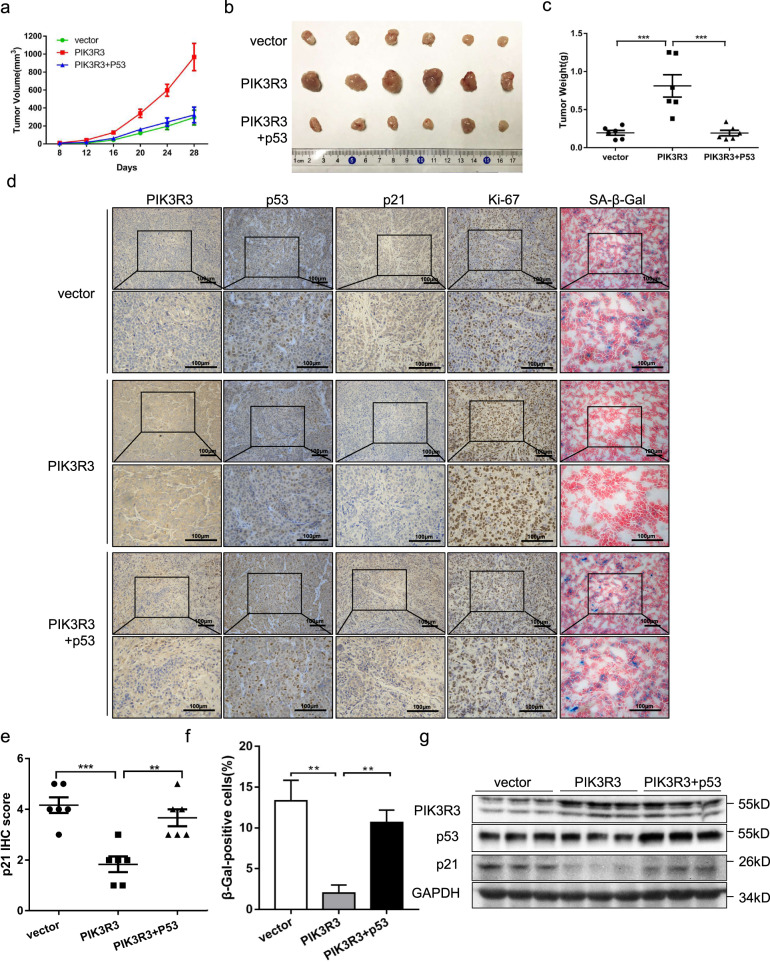
Overexpressed PIK3R3 promotes cell proliferation and inhibits cellular senescence in vivo. **a** Tumor volume of three groups were measured over time. **b** Tumor gross observation of three groups (vector, PIK3R3, and PIK3R3 + p53). **c** Tumor weight of three groups (vector, PIK3R3, and PIK3R3 + p53). **d** IHC assays of PIK3R3, p53, p21, Ki-67, and β-Gal from three groups. **e** Quantification of p21 expression from three groups. **f** Quantification of the proportion of β-Gal-positive cells from three groups. **g** WB of PIK3R3, p53, and p21 in three different groups. These numeral results were displayed as mean ± SD, *p < 0.05, **p < 0.01, ***p < 0.001, *t*-test.

We also injected three stable expressed cell lines (LoVo cells, respectively, transfected with negative control vector, PIK3R3 plasmid and PIK3R3 plasmid + p53 plasmid) into the subcutaneous tissues of nude mice (each group six mice, one million cells every mouse). After 1 week we measured the volume of the tumor every 4 days. A plotted tumor volume growth curve showed that the tumors of the PIK3R3 group grew much faster than the control group, but the PIK3R3 + p53 group grew more slowly than the PIK3R3 group (Fig. 6a). After the tumors grew for 1 month, we sacrificed all the nude mice and took out the tumor tissues. The tumor weights chart gave us an expected similar outcome (Fig. 6b, c). The IHC revealed that the proportion of Ki-67 staining cells in the PIK3R3 group was higher than that in the vector group, and in the PIK3R3 + p53 group was lower than that in the PIK3R3 group, but the proportion of β-Gal staining cells was exactly opposite to the above results (Fig. 6d, f). The p21 expression level of the PIK3R3 group decreased compared with the vector group, and increased in the PIK3R3 + p53 group compared with the PIK3R3 group (Fig. 6d, e). The staining level of HP1α and Lipofuscin (SBB staining) decreased in the PIK3R3 group compared with the vector group, and increased in the PIK3R3 + p53 group contrast to the PIK3R3 group (Fig. S5b). The protein levels of PIK3R3, p53, and p21 in three different groups were measured by western blotting, and we obtained the results similar to the data in vitro (Fig. 6g).

Overexpressed PIK3R3 promoted cell proliferation and suppressed cell senescence of subcutaneous xenograft tumor tissues in the mice model, which could be reversed by p53 overexpression. These results demonstrate that PIK3R3 plays an important role in the proliferation and cellular senescence of colorectal tumor cells in vivo in a p53-dependent manner.

## Discussion

CRC is one of the most common malignancies around the world with the complex genetic and biochemical background. The formation and progression of CRC is a multi-step, multi-stage, and multi-factor complex process, which is the result of interaction between the internal demic factors and the external environmental factors. Under the stimulation of chronic intestinal inflammation and intestinal flora, the activation of oncogenes and the inactivation of tumor suppressor genes promote the formation and progression of CRC^25,26^. The molecular pathogenesis of CRC is very intricate and multitudinous. Studying the molecular mechanisms of this cancer has important clinical implications, because they are associated with prognosis and treatment effects of the patients^27,28^. Therefore, the molecular mechanism of the development of CRC has important social significance for improving the survival rate and prognosis of CRC.

Previous studies have shown that PIK3R3 could participate in the regulation of various biological behaviors of tumor cells, such as proliferation, cell cycle, apoptosis, cell differentiation, invasion, and metastasis^10,29,30^. It was published that PIK3R3 is related to cell proliferation through different signaling pathways, but the interaction between PIK3R3 and p21 remains unknown. In our study, we found that the expression of PIK3R3 was significantly higher in human CRC tissues than in normal intestinal tissues, while the expression of p21 was significantly lower in human CRC tissues than in normal intestinal tissues. As we know, p21 is a cyclin-dependent kinase inhibitor regulated by p53 (ref. ^31^). We further verified that PIK3R3 could affect cell senescence in CRC cell lines, namely down-regulation of PIK3R3 significantly increased cell senescence of LoVo and SW48, while up-regulation of PIK3R3 can reduce senescence. Then through reverse experiments, namely p53 or p21 depletion/overexpression, we demonstrated that PIK3R3 relied on the p53/p21 signal pathway to regulate cell senescence.

Since p53 has the functions to inhibit cell proliferation and promote apoptosis, and the activity of p53 plays a key role in the success of traditional chemotherapy, many drugs causing DNA damage exert anti-tumor effects through p53-mediated apoptosis^32–35^. In the current study, we found that PIK3R3 can influence the transcriptional regulation of p21 by binding to p53, thereby regulating cellular proliferation and senescence. However, PIK3R3 did not show any effects on the transcription or degradation of p53. In other words, PIK3R3 did not affect the level of p53 protein but regulates its transcriptional regulatory activity of p21 gene. Then we found that PIK3R3 inhibited p53 binding to the p21 promoter region through interaction with p53, thus affected cell senescence and cell growth. The PIK3R3/p53/p21 axis acts as an important role in the regulation of cell senescence and proliferation in CRC.

In the studies of CRC tumor mechanism, the molecular regulation of cell senescence pathway has become more and more widely concerned. Although current published studies have only partially clarified the tendency of tumor treatment, p53 still plays a key role in its regulation. In the context of targeted tumor therapy, the ability of p53 to regulate the senescence of tumor cells is becoming an effective clinical approach to eliminate cancer cells in CRC. We can expect that the PIK3R3/p53/p21 axis will play an important role in the CRC treatment in the future.

In addition, we found that PIK3R3 played an important role in the SASP of LoVo and SW48 cell lines. As we know, SASP has been proved to be extremely important for cell senescence, and even tumor proliferation, invasion, and metastasis^19^. The specific regulatory mechanism of SASP has not been elucidated in previous studies. The elucidation of the PIK3R3/p53/p21 pathway could provide new research ideas for furthers studies on SASP as well as potential therapeutic options for CRC.

## Conclusions

In summary, we found that PIK3R3 acts as oncogene in CRCs in contrast to p21. High expression of PIK3R3 inhibits cell senescence and promotes cell proliferation, which is mediated by down-regulation of p21 transcription activity. This study suggests that the PIK3R3/p53/p21 signal pathway could be a potential target for clinical treatment of CRC.

## Materials and methods

### Clinical tumor samples

All studies including clinical samples were approved by Tongji Hospital Ethics Committee. The tumor tissues and corresponding normal intestinal tissues were acquired from CRC patients undergoing radical surgery in Wuhan Tongji Hospital (Hubei Province, PRC), 2017/04-2018/04. The above patients have signed informed consent before the operations. All patients did not receive neoadjuvant therapy before surgery.

### Cell lines, cell culture

The CRC cell lines (SW48 and LoVo) were obtained from the American Type Culture Collection (ATCC, Manassas, VA, USA). All cells were cultured at 5% CO_2_ and 37 °C in Dulbecco’s modified Eagle’s medium including 10% fetal bovine serum.

### Antibodies and reagents

The antibody to PIK3R3 (#11889) and HP1α (#2616) were purchased from Cell Signaling Technology Company (MA, USA). Antibodies to p53 (sc-47698) and p21 (sc-397) were purchased from Santa Cruz Company. Antibody to GAPDH (BM3876) was obtained from Wuhan Boster Company. Doxorubicin (Dox) was acquired from Calbiochem Company. Cycloheximide (CHX) was purchased from MedChemExpress Company. Cell Counting Kit-8 (CCK8) was gained from Wuhan Promoter Company. Lipofectamine 2000 was purchased from Invitrogen Company.

### Cell proliferation assay

Cell proliferation was measured through CCK8 assays. CRC cells were distributed into 96-well plates (4000 cells each well), and then incubated under the same conditions. CCK8 solution was piped into different wells and incubated for 1 h. The absorbance of every well was detected at a wavelength of 450 nm (*A*_450_).

### Cell cycle assay

After treating with 80% ethanol at −20 °C for several hours, cells was stained with propidium iodide at room temperature for 30 min. DNA content was detected through Becton-Dickinson FACScan System.

### β-Galactosidase staining

Cells were treated with Dox (0.25 μmol/l) and cultured at 5% CO_2_ and 37 °C for 48 h, and stained with SA-β-Gal staining working solution (Beyotime Biotechnology Ltd). SA-β-Gal staining cells of all cells in three indiscriminately chosen fields were counted through a light microscope.

### Western blot analysis and immunoprecipitation assay

The treated cells were lysed with NP40 on ice for 15 min. The lysate was centrifuged in 12,000 r.p.m. 4 °C for 10 min, and protein concentration of the supernatant was measured with the bicinchoninic acid assay kit (Thermo Fisher Scientific). The proteins were separated in SDS-PAGE by electrophoresis, and then transferred to the PVDF membrane afterwards. We incubated PVDF membranes with specified antibodies and tested them with chemiluminescent solution. For immunoprecipitation assay, whole-cell lysate was incubated overnight with specific antibodies, and then protein A/G beads were added for rotating 2~4 h at 4 °C. The beads were mixed with SDS sample buffer after three times washing with NP40 solution. The binding relationship of different proteins was confirmed by western blot.

### Immunofluorescence assay

The cell slides were washed in the culture plate three times with phosphate-buffered saline (PBS) for 3 min each. The slides were fixed with 4% paraformaldehyde for 15 min, and then were washed three times with PBS for 3 min each. 0.5% Triton X-100 (prepared with PBS) was used at room temperature for 20 min. After washing with PBS, 5% BSA was added on the slides, at room temperature for 30 min. We incubated cell slides with specified antibodies and acquired images with a confocal laser scanning microscope.

### Quantitative real-time PCR assay

Total mRNA was extracted with TRIzol (Invitrogen). And cDNA was generated through reverse transcription with PrimeScript RT Master Mix (Takara Biotechnology). Real-time PCR was performed with SYBR Green Real-time PCR Master Mix (TOYOBO) using Applied Biosystem 7300 (Thermo Fisher Scientific). The used primers are listed as follows: PIK3R3, 5′-ATGTACAATACGGTGTGGAGTATG-3′ (sense) and 5′-GCTGGAGGATCCATTTCAAT-3′ (anti-sense); p21, 5′-TGTCCGTCAGAACCCATGC-3′ (sense) and 5′-AAAGTCGAAGTTCCATCGCTC-3′ (anti-sense); p53, 5′-CAGCACATGACGGAGGTTGT-3′ (sense) and 5′-TCATCCAAATACTCCACACGC-3′ (anti-sense); GAPDH, 5′-GGAGCGAGATCCCTCCAAAAT-3′ (sense) and 5′-GGCTGTTGTCATACTTCTCATGG-3′ (anti-sense); CCL20 5′-TGCTGTACCAAGAGTTTGCTC-3′ (sense) and 5′-CGCACACAGACAACTTTTTCTTT-3′ (anti-sense); IL1β 5′-TGCACGCTCCGGGACTCACA-3′ (sense) and 5′-CATGGAGAACACCACTTGTTGCTCC-3′ (anti-sense); IL6 5′-TTCTGCGCAGCTTTAAGGAG-3′ (sense) and 5′-AGGTGCCCATGCTACATTTG-3′ (anti-sense); IL8 5′-ATGACTTCCAAGCTGGCCGTG-3′ (sense) and 5′-TGTGTTGGCGCAGTGTGGTC-3′ (anti-sense).

### IHC analysis

Two pathologists performed simultaneous IHC analysis with the light microscope to measure the staining situation of each sample. IHC staining was measured for quantification through the IRS system. The percent of positively stained cells was calculated with the following scores: 1 (<10%), 2 (10–50%), 3 (50–75%), and 4 (>75%). The scores of staining intensity were 0–3: 0, no staining; 1, light yellow; 2, yellow brown; 3, brown. The staining indexes were calculated by multiplying percent-score and intensity-score.

### Luciferase reporters assay

Tumor cells were co-transfected with luciferase reporter plasmids (contained p21 promoter construct) and TK-Renilla expression plasmids for 48 h. Luciferase activity was measured by the dual luciferase assay kit (Promega) according to the protocol. All experiments were repeated three times.

### ChIP assay

CRC cells (1 × 10^7^) were collected and afterwards fixed using 1% methanal for 10 min. Ultrasonic waves were used to break chromatin into fragments in the length of 200–1000 bp. The supernatant was incubated with specified antibody overnight. And protein A/G beads were added into the mixture and rotated 6 h at 2–4 °C. These beads were washed by PBS in order to elute the binding elements. The crosslinks of proteins and DNA fragments were reversed at 65 °C for 4 h. DNA fragments were treated with qPCR to analyze the binding levels of p53 to p21 promoter site. The ChIP primer sequences were listed as follows: p21 promoter 5′-TGGACTGGGCACTCTTGTCC-3′ (sense), 5′-CAGAGTAACAGGCTAAGGTT-3′ (anti-sense).

### Knockdown assay

Short interfering RNA (siRNA) of PIK3R3 and p53 were purchased from RiboBio Company. The sequences of PIK3R3 and p53 are listed below.

siPIK3R3 #1: 5′-GGACUUGCUUUAUGGGAAA-3′.

siPIK3R3 #2: 5′-AAGCUUUGGACAACCGAGAAA-3′.

sip53: 5′-GACUCCAGUGGUAAUCUAC-3′.

Sip21: 5′-ACACAAACUGAGACUAAGGCA-3′.

### Animal experiment

Cancer cells were suspended with Matrigel (BD Biosciences) (mixed with culture medium). Different groups of nude mice were injected with the appropriate cells (1 × 10^5^ cells/mouse). Tumor sizes were measured every 4 days. After 4 weeks of injection, all the nude mice were sacrificed. The tumor weight was measured.

### Statistical analysis

All statistical analyses were carried out with SPSS 24.0 (SPSS Inc.), and numeral results were conducted as mean ± SD. The difference between two groups was evaluated with two-tailed Student’s *t*-test (*p < 0.05, **p < 0.01, ***p < 0.001).

## Supplementary information

Supplementary figure legends

Supplementary figure 1

Supplementary figure 2

Supplementary figure 3

Supplementary figure 4

Supplementary figure 5
