# Supplementary material for: PIK3R3 inhibits cell senescence through p53/p21 signaling
Source: Cell Death Dis. 2020 Sep 24;11(9):798. doi: 10.1038/s41419-020-02921-z (PMC7519147; doi:10.1038/s41419-020-02921-z)
Supplement: Supplementary file 1 — Supplementary figure legends [file 41419_2020_2921_MOESM1_ESM.docx]

**Supplementary Figure Legends**

Figure S1. Knockdown of PIK3R3 promotes SASP in LoVo and SW48 cell lines. **a** and **b** qPCR assays of CCL20, IL1β, IL6 and IL8 mRNA in LoVo or SW48 cells with negative control/PIK3R3-knockdown after treatment of Dox(0.25μM, 48h). Every assay repeated 3 times, and these numeral results were displayed as mean ± SD, * p < 0.05, ** p < 0.01, *** p < 0.001, t-test.

Figure S2. PIK3R3 interacts with p53 protein and inhibits the nuclear accumulation of p53. **a** Immunofluorescent staining of 293T cells transfected with His-p53 or His-p53+Flag-PIK3R3 via His and Flag antibodies. DAPI was used as a nuclear dye. Every assay repeated 3 times. **b** and **c** Western blot analysis of PIK3R3, p53, Lamin AC (nuclear control) and GAPDH in the cytosolic and nuclear fractions of LoVo cells transfected with siPIK3R3(#1,#2) or PIK3R3 plasmid. Every assay repeated 3 times.

Figure S3. PIK3R3 regulates cell senescence through a p21-dependent pathway. **a** WB of PIK3R3, p21, HP1α and GAPDH in LoVo and SW48 cells transfected with siPIK3R3#1 or/and sip21. **b** WB of PIK3R3, p21, HP1α and GAPDH in LoVo and SW48 cells transfected with PIK3R3 plasmid or/and Flag-p21 plamid. Every assay repeated 3 times.

Figure S4. Mechanism model: PIK3R3/p53 interaction inhibits the binding of p53 protein to p21 gene promoter, resulting in suppression of p21 transcription, which induces cell senescence inhibition and cell growth promotion.

Figure S5. PIK3R3 affects cell senescence in vivo. **a** IHC assays of HP1α and Sudan Black B (SBB) staining from three groups (shNC, shPIK3R3 and shPIK3R3+shp53) in formalin-fixed paraffin-embedded (FFPE) sections. **b** IHC assays of HP1α and SBB staining from three groups (vector, PIK3R3 and PIK3R3+p53) in FFPE sections.
