# Supplementary figures and images for: PIK3R3 inhibits cell senescence through p53/p21 signaling
Source: Cell Death Dis. 2020 Sep 24;11(9):798. doi: 10.1038/s41419-020-02921-z (PMC7519147; doi:10.1038/s41419-020-02921-z)

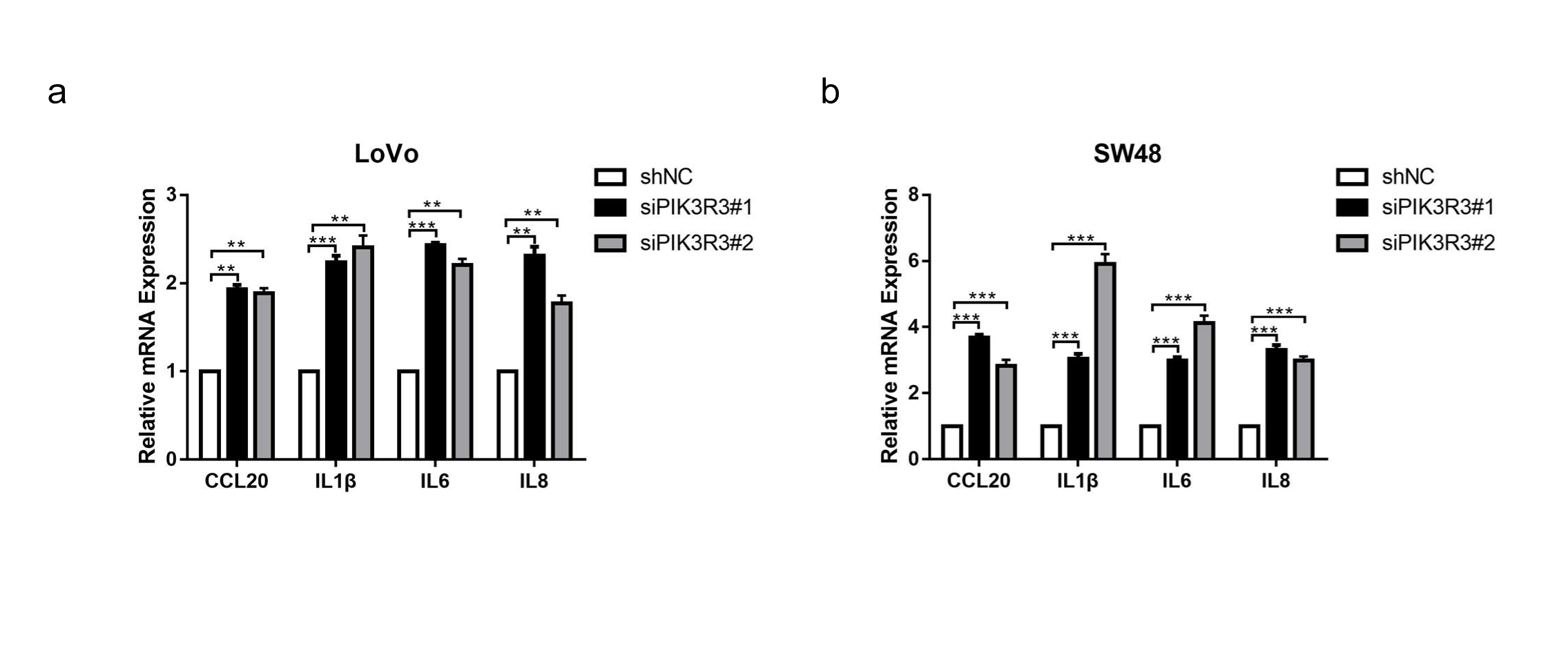

Supplement: Supplementary file 2 — Supplementary figure 1 [file 41419_2020_2921_MOESM2_ESM.tif]

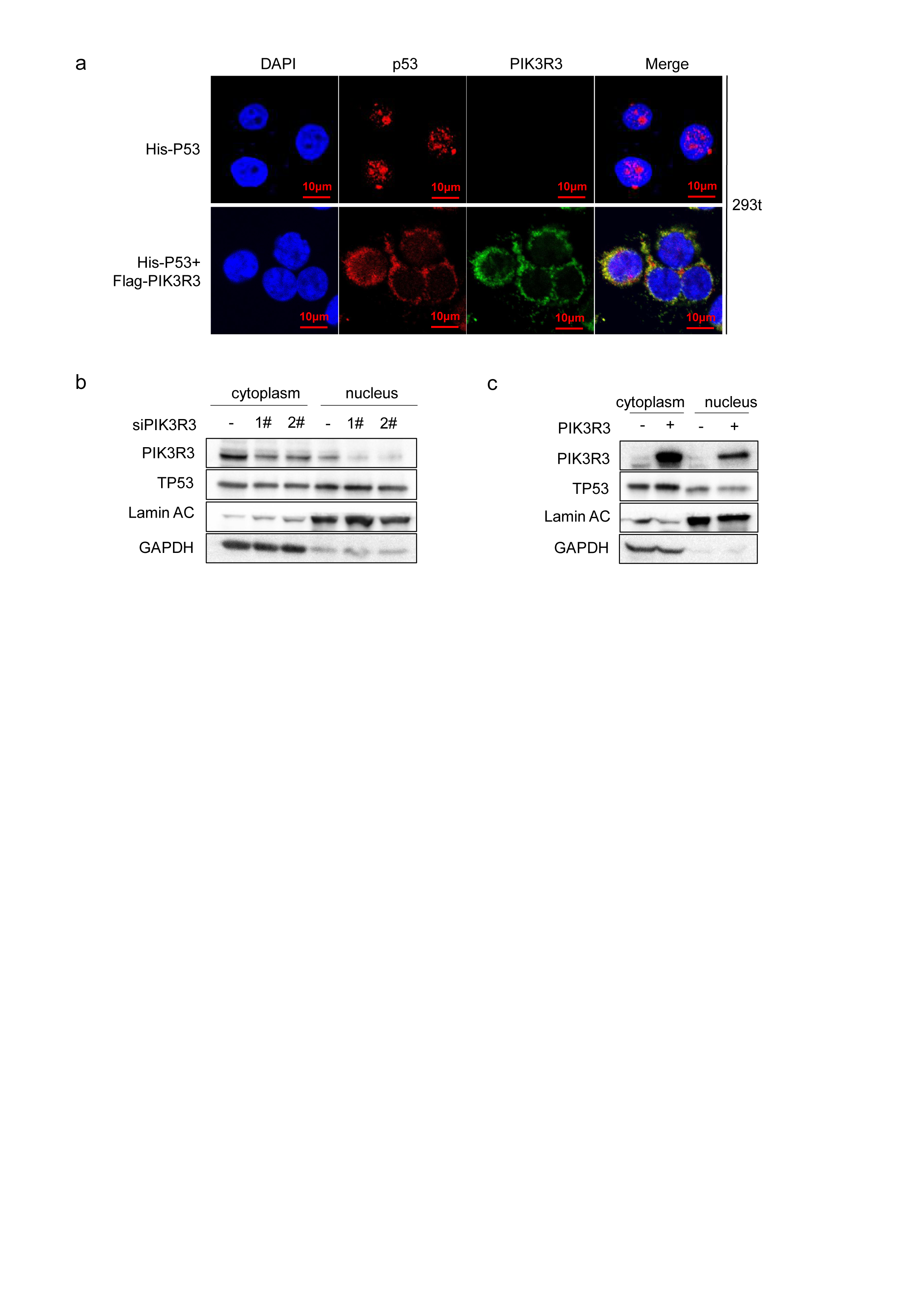

Supplement: Supplementary file 3 — Supplementary figure 2 [file 41419_2020_2921_MOESM3_ESM.tif]

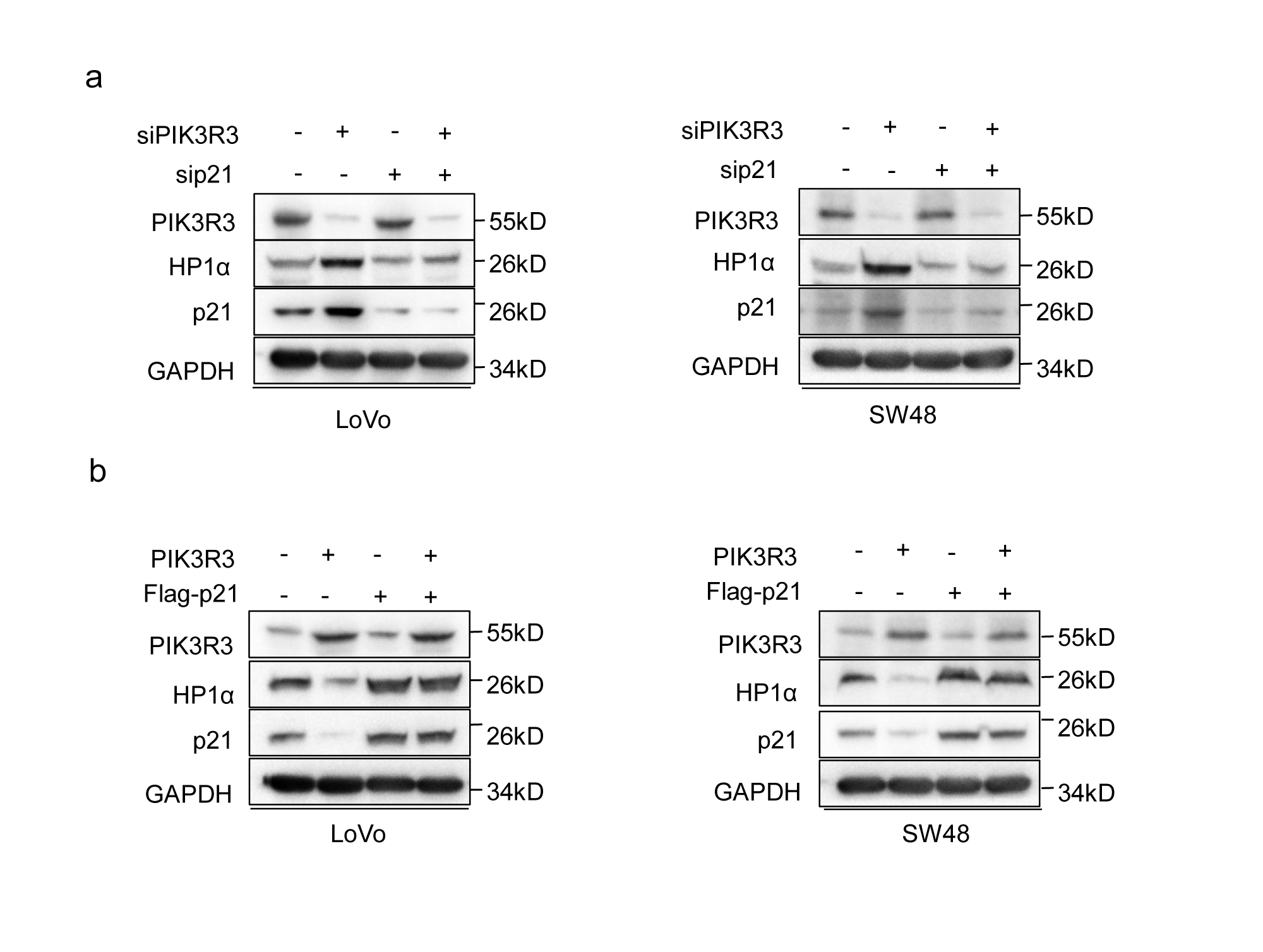

Supplement: Supplementary file 4 — Supplementary figure 3 [file 41419_2020_2921_MOESM4_ESM.tif]

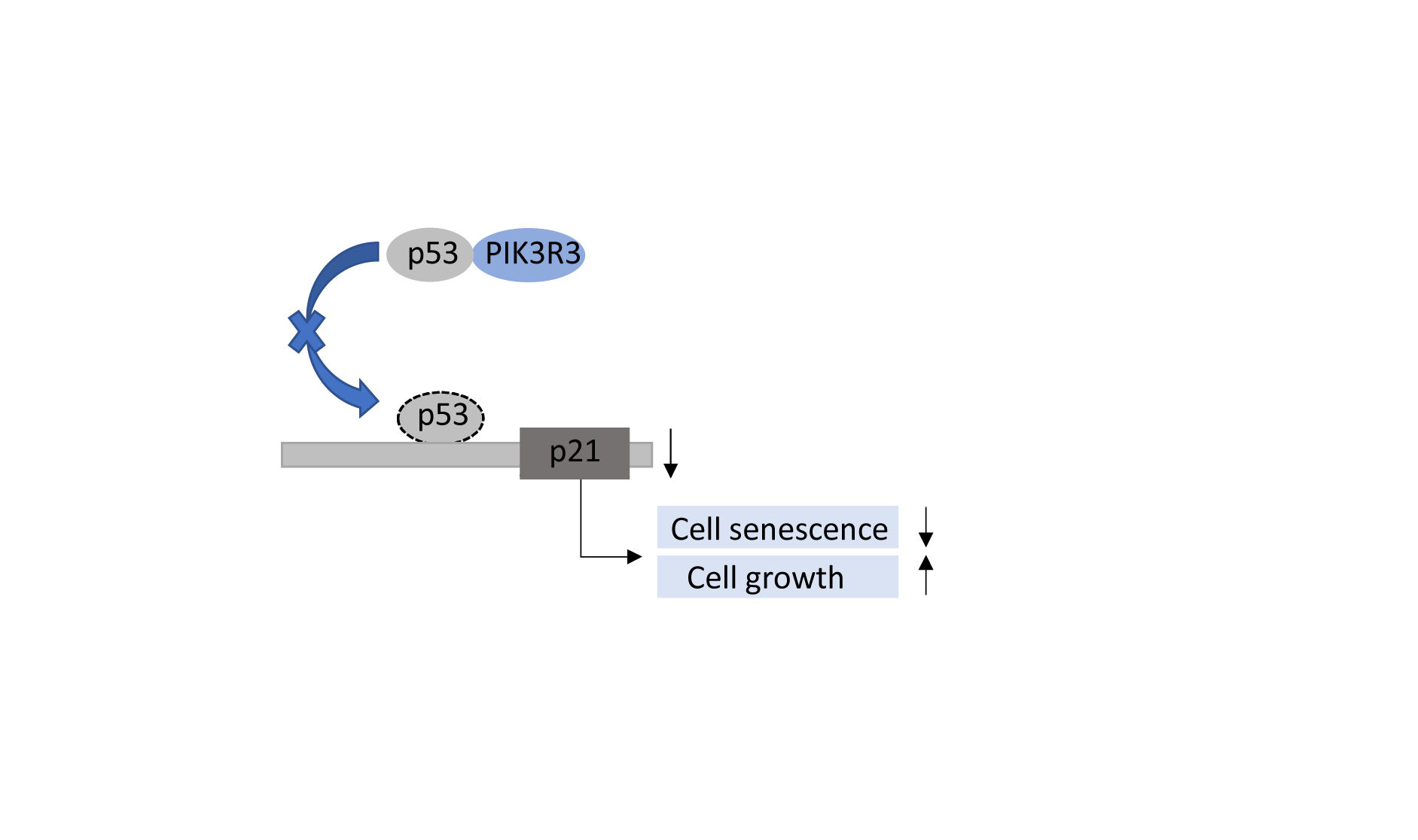

Supplement: Supplementary file 5 — Supplementary figure 4 [file 41419_2020_2921_MOESM5_ESM.tif]

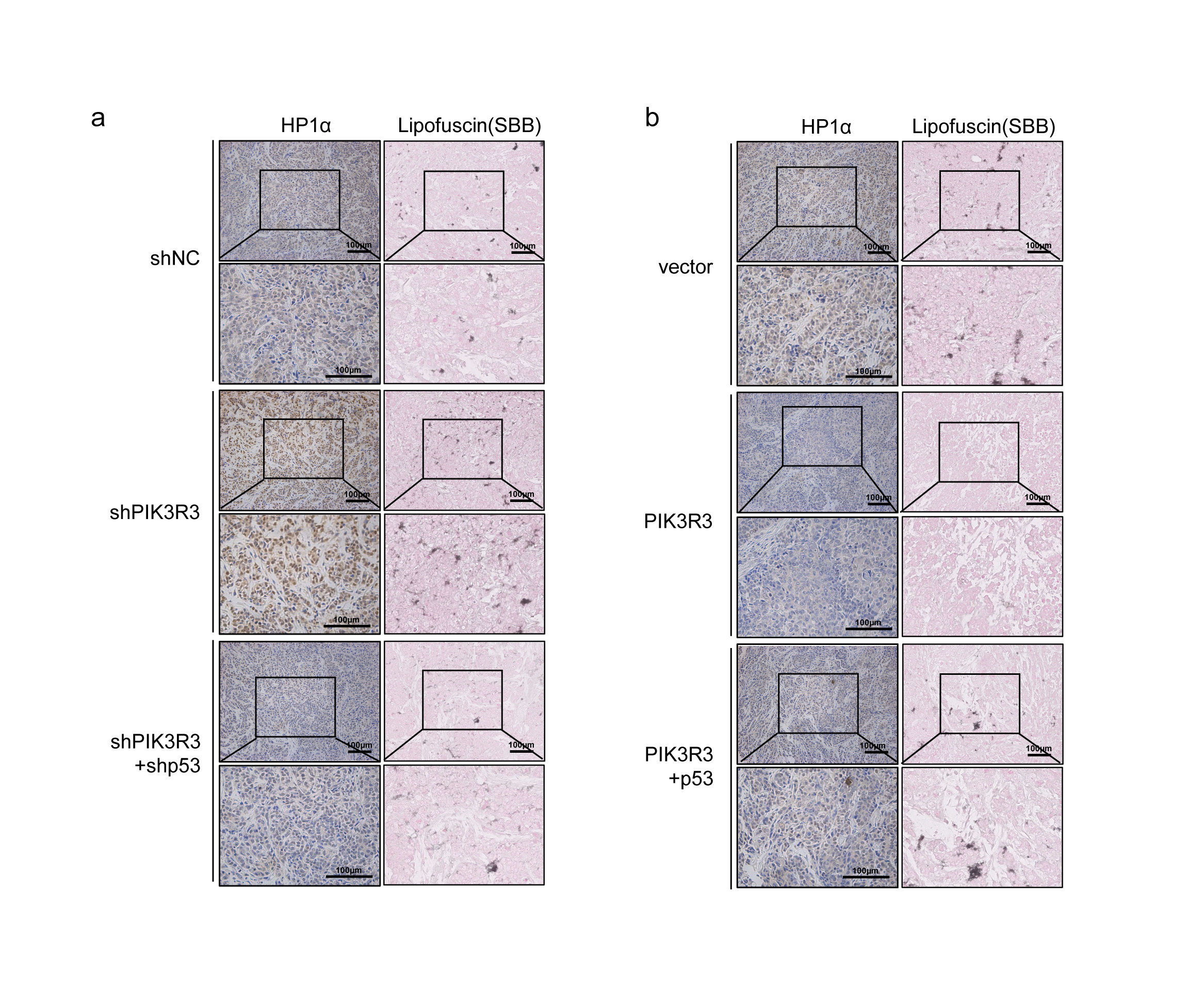

Supplement: Supplementary file 6 — Supplementary figure 5 [file 41419_2020_2921_MOESM6_ESM.tif]
